# IL-33 is induced in undifferentiated, non-dividing esophageal epithelial cells in eosinophilic esophagitis

**DOI:** 10.1038/s41598-017-17541-5

**Published:** 2017-12-14

**Authors:** J. Travers, M. Rochman, J. M. Caldwell, J. A. Besse, C. E. Miracle, M. E. Rothenberg

**Affiliations:** Division of Allergy and Immunology, Cincinnati Children’s Hospital Medical Center, Cincinnati, Ohio 45229-3039 USA

## Abstract

The molecular and cellular etiology of eosinophilic esophagitis (EoE), an emerging tissue-specific allergic disease, involves dysregulated gene expression in esophageal epithelial cells. Herein, we assessed the esophageal expression of IL-33, an epithelium-derived alarmin cytokine, in patients with EoE. IL-33 protein was markedly overexpressed within the nuclei of a subpopulation of basal layer esophageal epithelial cells in patients with active EoE compared to control individuals. IL-33 exhibited dynamic expression as levels normalized upon EoE remission. IL-33–positive basal epithelial cells expressed E-cadherin and the undifferentiated epithelial cell markers keratin 5 and 14 but not the differentiation marker keratin 4. Moreover, the IL-33–positive epithelial cells expressed the epithelial progenitor markers p75 and p63 and lacked the proliferation markers Ki67 and phospho-histone H3. Additionally, the IL-33–positive cells had low expression of PCNA. IL-33 expression was detected in *ex vivo*–cultured primary esophageal epithelial cells in a subpopulation of cells lacking expression of proliferation markers. Collectively, we report that IL-33 expression is induced in an undifferentiated, non-dividing esophageal epithelial cell population in patients with active EoE.

## Introduction

Eosinophilic esophagitis (EoE) is an emerging chronic, food antigen-driven, inflammatory allergic disorder^1^. It is notable for type 2 inflammation associated with esophageal structural changes and infiltration of immune cells rich in eosinophils into the esophageal epithelium^2^. There is a critical need to identify which factors initiate and propagate the excessive immune responses against food antigens in EoE.

The innate cytokine interleukin 33 (IL-33) is a prominent potentiator of type 2 immunity^3^. IL-33 is generally expressed within nuclei of mucosal epithelial cells, fibroblasts, and endothelial cells^4^. Classically, IL-33 acts as an alarmin, as it is released extracellularly following cellular necrosis and can activate a wide variety of immune cells that express its plasma membrane receptor, suppressor of tumorigenicity 2 (ST2). Importantly, IL-33 is a very potent activator of eosinophils^5^, mast cells^6^, basophils^7,8^, and type 2 innate lymphoid cells (ILC2)^9^, which all infiltrate the esophagus in patients with EoE^1,10–12^. Furthermore, intraperitoneal injection of recombinant IL-33 induces esophageal responses that mimic EoE, including eosinophil infiltration, increased proliferation of esophageal epithelial cells, and production of type 2-associated cytokines^13^. In addition, mice genetically deficient in *IL33* or *IL1RL1* (encodes ST2) have attenuated ovalbumin-induced EoE-like disease^14,15^. These findings are likely to be clinically relevant because there is association between genetic variants in the *IL33* locus and EoE disease risk^16^, as well as with blood eosinophilia^17^. Herein, we report that patients with active EoE have markedly increased detection of IL-33 present in the nuclei of esophageal basal layer cells with high levels of E-cadherin, p75, p63, and keratins (KRT) 5 and 14 and low expression of proliferating cell nuclear antigen (PCNA). These IL-33–positive basal layer cells lack KRT4, Ki67, and phospho-histone H3. Levels of IL-33 normalize to undetectable levels following disease remission. Examining primary esophageal epithelial cell cultures *ex vivo*, IL-33 was detectable in a subpopulation of cells lacking expression of proliferation markers. Collectively, we propose that IL-33 is induced in an undifferentiated, mitotically inactive esophageal epithelial population in patients with EoE.

## Results

### IL-33 expression in eosinophilic esophagitis

**Figure 1:**
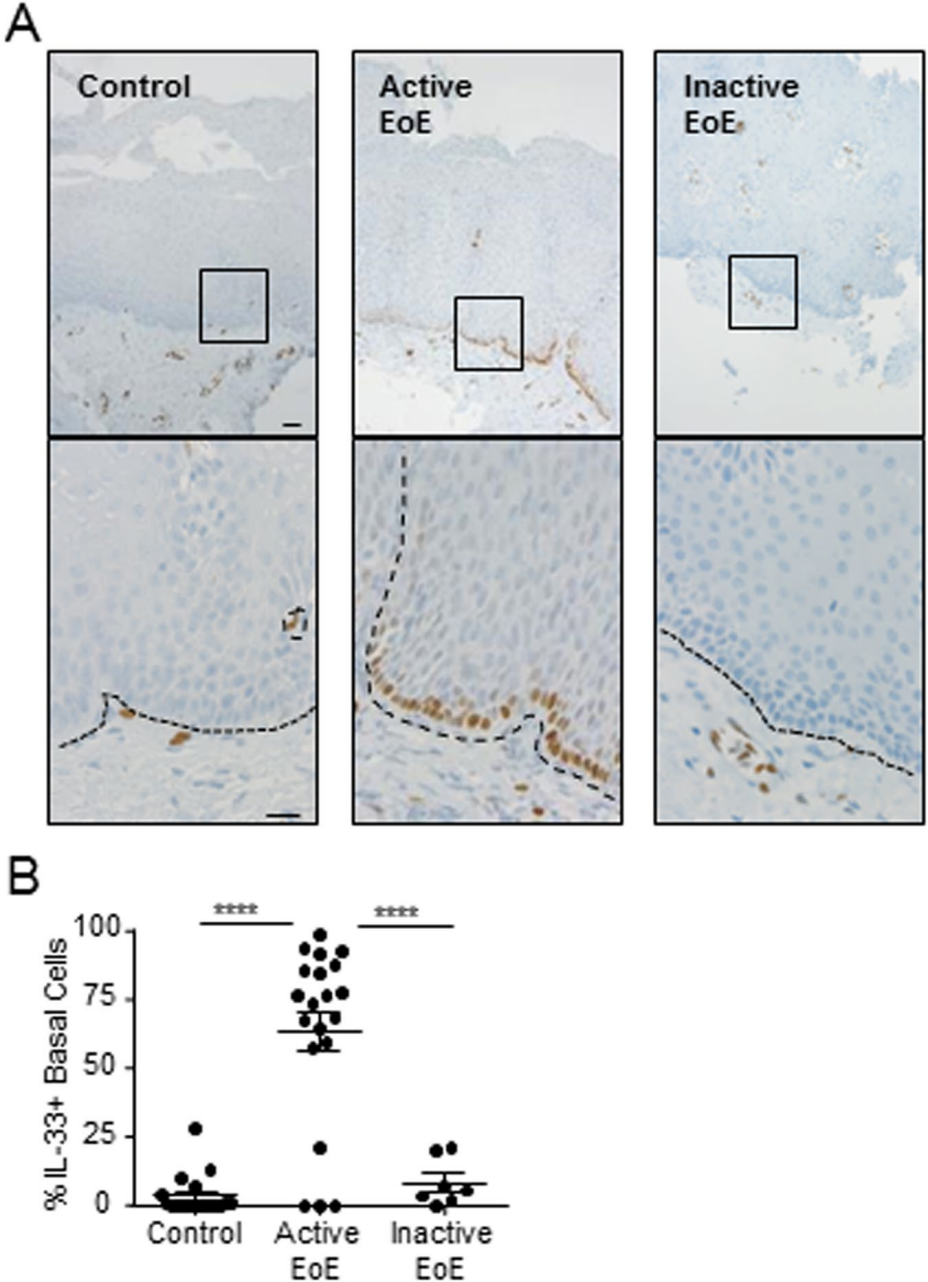
Subcellular localization of IL-33 in esophageal epithelial cells. (**A**,**B**) Immunohistochemistry for IL-33 protein expression in representative esophageal biopsies from healthy control individuals (Control, left panel), patients with active EoE (Active EoE, middle panel), or patients with inactive EoE (Inactive EoE, right panel) using mouse anti–IL-33 antibody. In (**A**), the bottom row is a high-power view of the area enclosed in the black square. The black dashed lines indicate the basement membrane. Scale bars are both 20 µm. Biopsies from 19 controls, 20 patients with active EoE, and 7 patients with inactive EoE were stained. (**B**) Quantification of the proportion of basal layer cells in each biopsy with IL-33 expression. Mean ± standard error of the mean is depicted. ****p < 0.0001.

We assessed IL-33 protein expression by immunohistochemistry in esophageal biopsies from patients with active or inactive EoE and from normal controls. In the healthy esophagus, IL-33 protein expression was detected in lamina propria cells, including endothelial cells (Fig. 1A). However, there was little expression of IL-33 within the homeostatic esophageal epithelium (Fig. 1A). In contrast, there was a substantial increase of IL-33 within the epithelium in esophageal biopsies of patients with active EoE (Fig. 1A,B). Almost all IL-33 expression within the epithelium was limited to a subpopulation of basal layer cells within the interpapillary basal layer (IBL) (Fig. 1A). There was a significant increase in the average percentage of basal layer cells with detectable IL-33 expression in biopsies from active EoE patients compared to control individuals (64 ± 7% vs 4 ± 2% [mean ± SEM], p < 0.0001) (Fig. 1B). We next aimed to determine whether esophageal IL-33 levels within the epithelium were constitutively high in patients with EoE regardless of disease remission status. To test this, we compared the esophageal IL-33 expression within the epithelium in patients with active and inactive EoE, defined as patients with a history of EoE and ≤1 eosinophils per high-power field of esophageal biopsy. IL-33 levels normalized with disease remission (Fig. 1B). We were struck by the finding that IL-33 was restricted to nuclei as no cytoplasmic or extracellular staining was observed. These results demonstrate that IL-33 is induced within the esophageal epithelium in patients with active EoE as compared to individuals without ongoing allergic inflammation.

### Characterization of IL-33–positive basal layer cells *in vivo*

**Figure 2:**
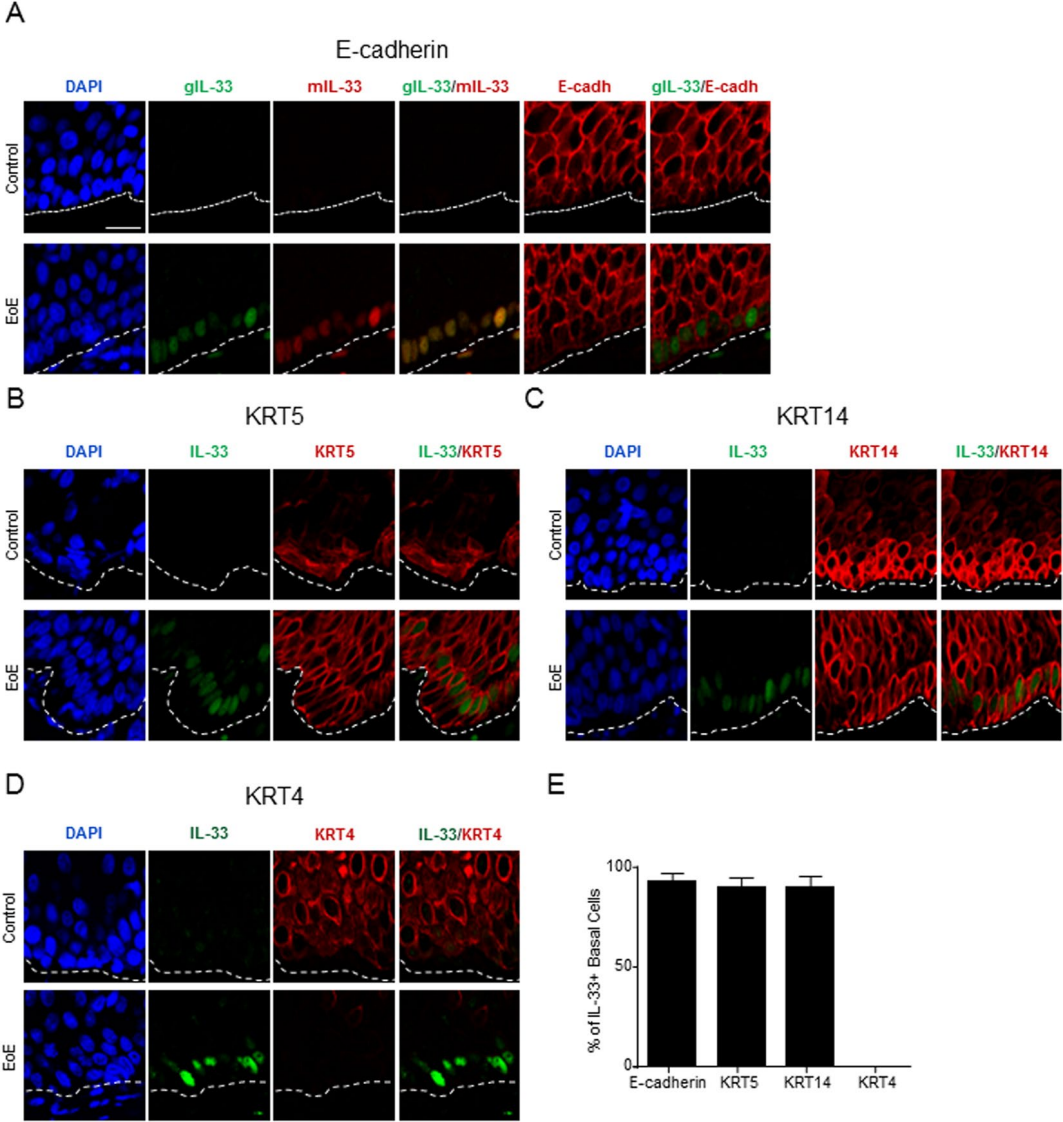
Epithelium-specific protein expression in esophageal tissue. (**A**–**D**) Immunofluorescence of esophageal biopsies from control individuals (top row) or patients with active EoE (bottom row). Nuclei are indicated by DAPI staining (blue). Green and red indicate staining with the indicated antibodies. The white dashed lines indicate the basement membrane. Scale bar is 20 µm. Images are representative of biopsies from 4 or 5 patients with active EoE and 4 or 5 control individuals. (**E**) Quantification of the proportion of IL-33–positive basal layer cells from active EoE biopsies with strong expression of the indicated marker from (**A**–**D**). Mean ± standard error of the mean is depicted. E-cadh, E-cadherin; DAPI, 4′,6-diamidino-2-phenylindole; gIL-33, goat anti–IL-33 antibody; mIL-33, mouse anti–IL-33 antibody; KRT, keratin.

To further characterize IL-33–expressing cells, we assessed IL-33 expression in esophageal biopsies by immunofluorescence using two different anti–IL-33 antibodies (a monoclonal antibody raised in mouse and a polyclonal antibody raised in goat). No staining was detected using either antibody within the epithelium in control individuals (Fig. 2A, low-power magnification images in Supplementary Figure 1). Strong staining with both antibodies was detected within the IBL in patients with active EoE (Fig. 2A). Notably, only nuclear expression was found as the staining from the anti–IL-33 antibodies overlapped with the DNA-binding dye 4′,6-diamidino-2-phenylindole (DAPI) (Fig. 2A). IL-33–positive cells had strong expression of the epithelial marker E-cadherin (Fig. 2A,E). Next, we assessed the differentiation status of the IL-33–positive IBL cells by co-staining with markers of different epithelial populations. There was increased expression of the undifferentiation markers KRT5 and KRT14^18^ and decreased expression of the differentiation marker KRT4 in biopsies from active EoE patients compared to biopsies from control individuals (Fig. 2B–D). IBL cells in biopsies from active EoE patients and control individuals expressed KRT5 and KRT14 (Fig. 2B,C,E) but not KRT4 (Fig. 2D,E). Collectively, these results demonstrate that IL-33 is induced in a population of undifferentiated epithelial cells in patients with active EoE.

**Figure 3:**
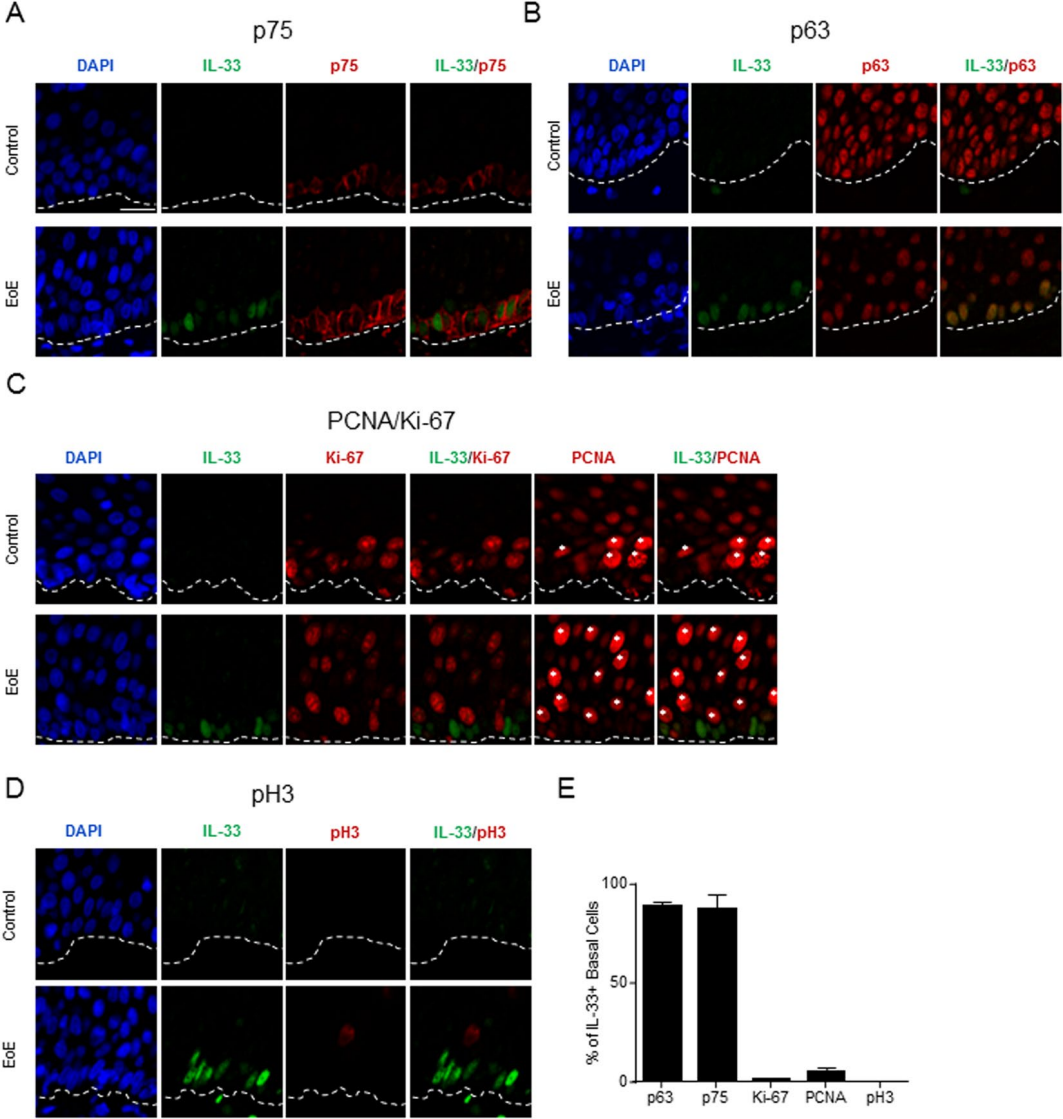
Cell cycle and differentiation status *in vivo* in esophageal tissue. (**A**–**D**) Immunofluorescence of esophageal biopsies from control individuals (top row) or patients with active EoE (bottom row). Nuclei are indicated by DAPI staining (blue). Green and red indicate staining with the indicated antibodies. The white dashed lines indicate the basement membrane. Scale bar is 20 µm. White asterisks indicate cells with high expression of PCNA. Images are representative of biopsies from 3–6 patients with active EoE and 3–6 control individuals. (**E**) Quantification of the number of IL-33–positive basal layer cells from active EoE biopsies with strong expression of the indicated marker from (**A**–**D**). Mean ± standard error of the mean is depicted. DAPI, 4′,6-diamidino-2-phenylindole; pH3, phospho-histone H3; PCNA, proliferating cell nuclear antigen.

Because esophageal epithelial progenitor cells exist within the basal layer of the homeostatic esophagus^19^, we hypothesized that the IL-33–expressing IBL cells within the esophagus of patients with active EoE constituted an epithelial progenitor population. IBL cells expressed the epithelial progenitor markers p75^20^ (Fig. 3A,E, low-power magnification images in Supplementary Figure 2) and p63^19^ (Fig. 3B,E) independently of EoE disease status. Next, the cell cycle status was assessed by performing immunofluorescence with a panel of proliferation markers. Consistent with previous reports indicating increased rates of proliferation within the esophageal epithelium of patients with active EoE^21,22^, there were increased number of cells positive for Ki-67^23^, phospho-histone H3^24^, and PCNA (Fig. 3C,D). In biopsies from both active EoE patients and control individuals, IBL cells did not express Ki-67 (Fig. 3C,E) or phospho-histone H3 (Fig. 3D,E) and only had low expression of PCNA (Fig. 3C), which is strongly upregulated during S phase^25^. These results indicate that these IL-33–positive basal layer cells express markers consistent with being a non-dividing epithelial progenitor population.

### Characterization of IL-33 expression *ex vivo*

**Figure 4:**
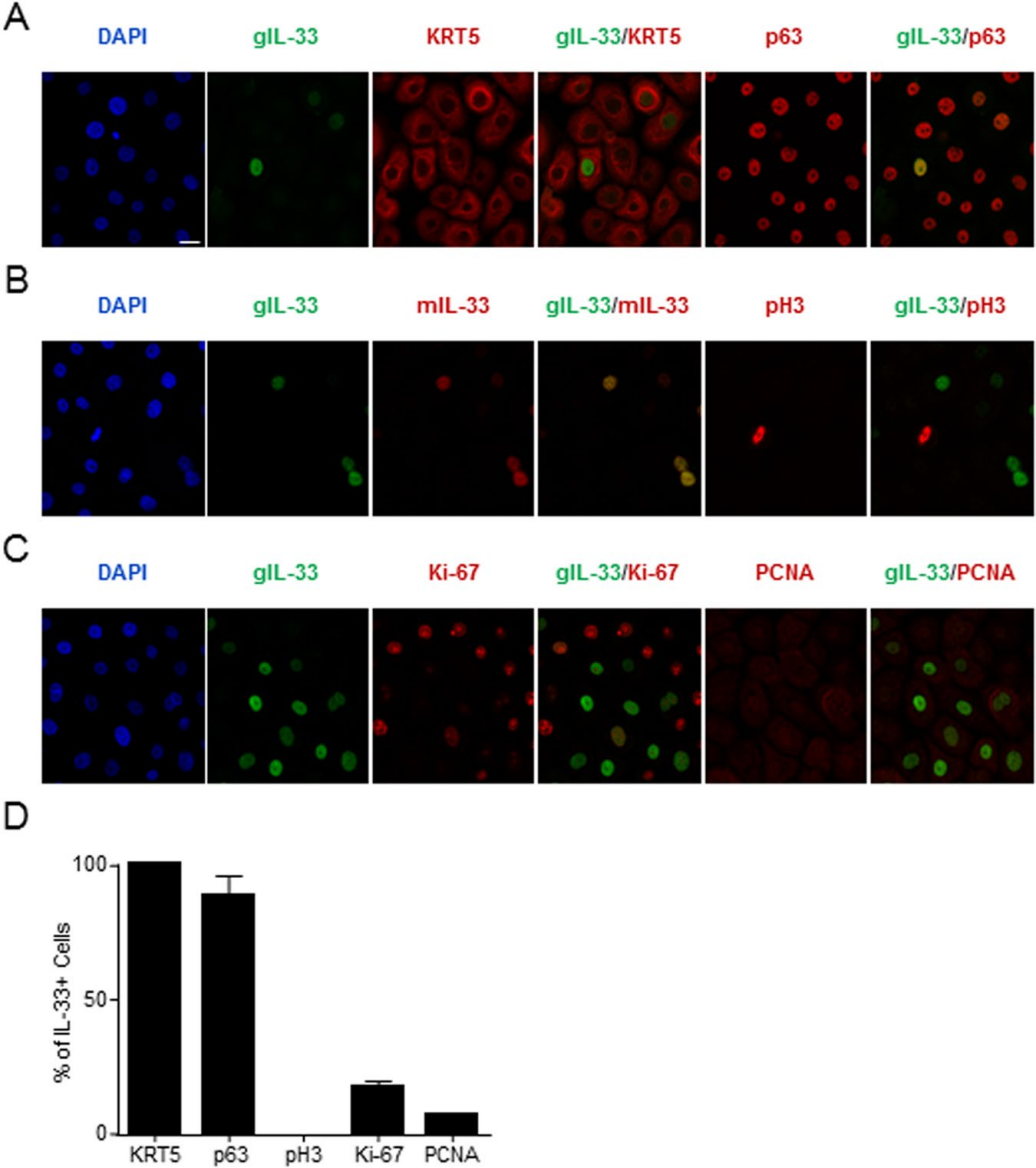
Cell cycle status of IL-33–expressing esophageal cells *ex vivo*. (**A**–**C**) Immunofluorescence of *ex vivo*–cultured primary esophageal epithelial cells. Nuclei are indicated by DAPI staining (blue). Green and red indicate staining with the indicated antibodies. Images are representative of three independent experiments. (**D**) Quantification of the percentage of IL-33–positive primary epithelial cells with strong expression of the indicated marker. Mean ± standard error of the mean of cumulative data from three independent experiments is depicted. Scale bar is 20 µm. DAPI, 4′,6-diamidino-2-phenylindole; gIL-33, goat anti–IL-33 antibody; mIL-33, mouse anti–IL-33 antibody; KRT, keratin; pH 3, phospho-histone H3; PCNA, proliferating cell nuclear antigen.

To determine whether restriction to a mitotically quiescent subpopulation of esophageal epithelial cells is an intrinsic feature of IL-33, we assessed IL-33 expression in *ex vivo* cultures of primary esophageal epithelial cells. Cells were maintained in an undifferentiated state as nearly all of the cells, including those with detectable IL-33 expression, were positive for KRT5 and p63 (Fig. 4A,D). Nuclear expression of IL-33 was detected using two independent anti–IL-33 antibodies in unstimulated cultures (Fig. 4B). Comparable intracellular levels of IL-33 were detected in cultures derived from both patients with active EoE and normal controls (data not shown). Additionally, no mitotic cells, defined by positive expression of phospho-histone H3, had detected expression of IL-33 using either antibody (Fig. 4B,D). Additionally, the vast majority of IL-33–positive cells lacked Ki-67 and had low expression of PCNA (Fig. 4C,D). In total, these results illustrate that IL-33 is expressed in a subpopulation of *ex vivo*–cultured esophageal epithelial cells that is not actively dividing.

We aimed to characterize the factors that regulate IL-33 expression in esophageal epithelial cells in the context of EoE. We first tested the effect of IL-13 because it is dramatically up-regulated in patients with EoE^26^, robustly reproduces the disease transcriptome^26^, and is a major driver of its pathogenesis^27^, as evidenced by the beneficial effects of humanized anti–IL-13 treatment in patients with EoE^28^. IL-13 treatment had no detectable effect on IL-33 expression in primary epithelial cells on both the mRNA and protein levels (Supplementary Figure 3A,B). We next examined oncostatin M (OSM), which has previously been shown to induce IL-33 expression in mouse lung alveolar cells^29^. Both OSM and its receptor exhibited increased expression within the esophagus of patients with EoE (Supplementary Figure 4A–C). Treatment of primary esophageal epithelial cells for 24 hours with OSM (100 ng/mL) increased IL-33 protein by approximately 66% (p < 0.001) as determined by Western blot (Supplementary Figure 4D,E). This suggests that the induction of IL-33 within the esophageal epithelium of patients with active EoE may be due at least in part to the action of OSM.

## Discussion

We have assessed esophageal expression of IL-33 in patients with active EoE. We report that (1) IL-33 protein expression is increased within the epithelium in patients with active EoE; (2) IL-33 expression is dynamically regulated as a function of disease activity as its expression normalizes upon disease remission; (3) IL-33 protein has a nuclear compartmentalization; (4) IL-33 is expressed in a subpopulation of basal layer cells; (5) IL-33–positive basal cells express the epithelial cell markers E-cadherin, KRT5, KRT14, p75, and p63; (6) IL-33–positive basal cells are primarily non-dividing cells as assessed by low levels of PCNA, Ki-67, and phospho-H3; and (7) IL-33 protein is detected in primary human esophageal epithelial cells in a sub-population lacking expression of proliferation markers.

**Figure 5:**
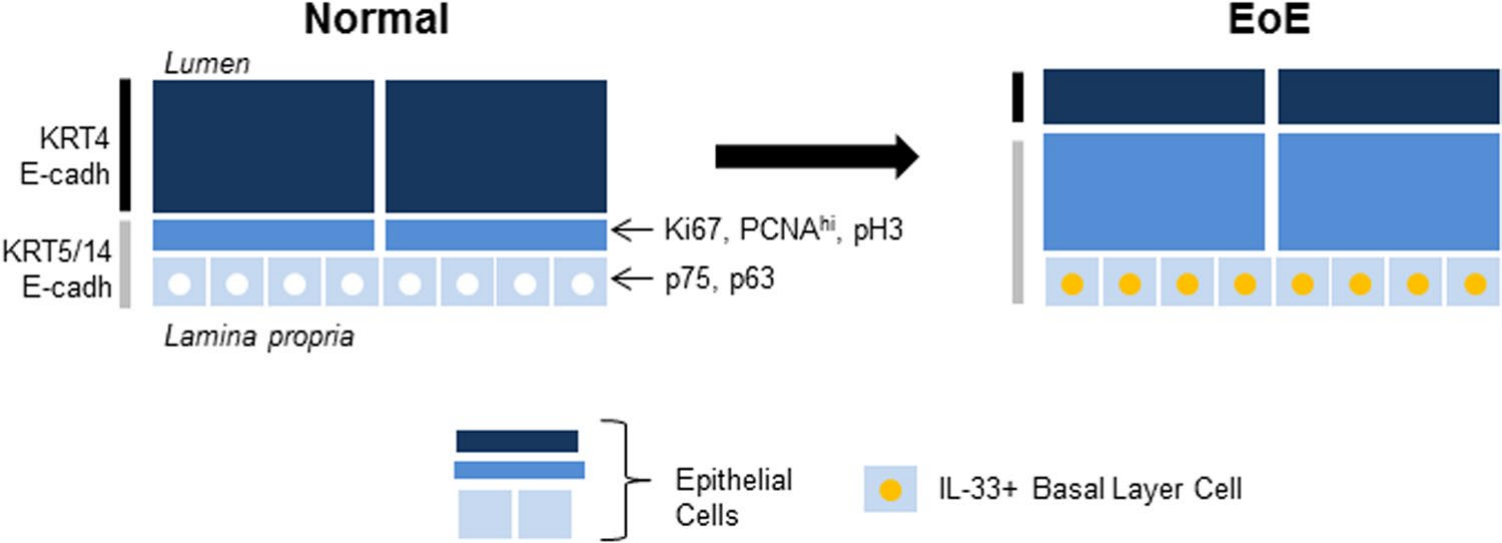
Summary of markers of IL-33–positive esophageal epithelial cells in EoE. In the esophagus of patients with active EoE, IL-33 (yellow circle) is induced in the nuclei of interpapillary basal layer cells. In both patients with EoE and healthy individuals, these basal layer cells express E-cadherin, undifferentiated keratins (KRT5, KRT14), and epithelial progenitor markers (p75, p63). IL-33^+^ cells do not express the differentiation marker KRT4 or the proliferation markers Ki67, pH 3, or PCNA. Gray vertical bar indicates epithelial cell layers expressing KRT5 and KRT14, and black vertical bar indicates those layers expressing KRT4. For clarity, papillae are not depicted. E-cadh, E-cadherin; EoE, eosinophilic esophagitis; IL, interleukin; KRT, keratin; PCNA, proliferating cell nuclear antigen; pH3, phospho-histone H3.

We identified IL-33 expression within the esophageal epithelium of patients with active EoE exclusively in basal layer cells (see model in Fig. 5). A previous report showed IL-33 expression only in the lamina propria^13^. There were not any apparent differences in the processing of esophageal biopsies between the two studies, and the same anti–IL-33 antibody was used. Perhaps Judd *et al*. did not observe IL-33 expression within the epithelium due to the orientation of the biopsies they used, which did not appear to have a clear interpapillary basal layer, which is where IL-33 expression was localized in our study. We also extend their findings by identifying that IL-33 levels within the esophageal epithelium normalize upon disease remission, indicating that the increased IL-33 expression is an acquired, rather than constitutive or intrinsic, feature of EoE.

It remains unclear why IL-33 protein expression within the esophageal epithelium is restricted to interpapillary basal layer cells *in vivo*. In light of the high potency of extracellular IL-33–induced activation of immune cells^4,5^, limiting the number of IL-33–expressing cells could serve to prevent excess release. Interestingly, the IL-33 expression profile in the esophageal epithelium is different than those of thymic stromal lymphopoietin (TSLP) and IL-25, which are expressed in superficial layers^30^. It is notable that interpapillary basal layer cells do not actively proliferate. IL-33 has been shown to be restricted to non-proliferating cells in other cell types, including stomach surface mucus cells^31^ and endothelial cells^32^. Quiescent endothelial cells are known to express IL-33 by a mechanism regulated by the Notch1 ligands Dll4 and Jagged1^33^. IFNγ is known to be a strong inducer of IL-33 expression in primary skin keratinocytes^34–36^. *Ex vivo* culture of primary esophageal epithelial cells offers a model system for future investigations regarding the regulation of IL-33 expression. We observed comparable IL-33 protein expression in non-proliferating primary esophageal epithelial cells derived from active EoE patients and healthy controls under baseline conditions (i.e. without stimulation with disease-relevant conditions) despite the fact that it was not expressed by esophageal keratinocytes in the homeostatic esophagus. This suggests that IL-33 was induced during *ex vivo* culture. Our results mirror other work showing detectable IL-33 expression in primary skin keratinocytes derived from healthy donors despite the fact that it was not expressed within the epidermis of healthy humans *in vivo* ^34^. Our results revealed that IL-33 expression in primary epithelial cell cultures was increased by OSM but was not affected by IL-13 treatment. It is likely that a combination of multiple factors is responsible for the induction of IL-33 in esophageal epithelial cells in EoE, so future investigation into the regulation of IL-33 expression is warranted.

Despite the observation of strong nuclear expression of IL-33 within the esophageal epithelium, IL-33 presumably needs to be released extracellularly in order to induce or propagate immune responses. The likely mechanism is passive release after cellular damage or necrosis, which is the classical mechanism of its release. In line with this, we observed detectable IL-33 in supernatants of primary esophageal epithelial cells after induction of necrosis but not after treatment with pro-allergic cytokines IL-13 or OSM (data not shown). Potential mediators of epithelial cell damage and/or necrosis include eosinophil granule proteins and proteases.

Our study characterizes the IL-33–expressing basal layer cells in EoE as a mitotically quiescent progenitor population. This supports both *ex vivo* spheroid culture studies demonstrating that the esophageal epithelial cells with the highest stem cell capacity are present in the basal layer^19^ and *in vivo* lineage tracing studies showing the existence of a long-lived progenitor population in basal cells^37^. EoE is a hyperproliferative disorder^22,38^ with marked loss of esophageal tissue identity and differentiation within the epithelium^39^. Because this cell layer purportedly undergoes occasional mitotic divisions in order to maintain the esophageal epithelium^40^, future studies should investigate their contributions to disease pathogenesis. IL-33 has long been proposed to act as a transcriptional regulator through its ability to bind chromatin^41,42^. No rigorously tested evidence for an intracellular nuclear function for IL-33 has been identified. However, the effect of nuclear IL-33 expression in these basal layer cells, especially in the context of allergic inflammation, has not been examined and thus warrants future investigation. Taken together, our data identified that IL-33 is induced in a non-dividing esophageal epithelial progenitor population in patients with active EoE. We also found that IL-33 was dynamically expressed as a function of disease activity. These findings underscore the potential value of further understanding the regulation and role of IL-33, in EoE and other allergic diseases.

## Methods

### Antibodies

Mouse monoclonal antibody against IL-33 (clone Nessy-1) (#ALX-804-840-C100) was purchased from Enzo. Rabbit polyclonal antibodies against KRT5 (#ab24647) and Ki-67 (#ab15580) were purchased from Abcam (Abcam, Cambridge, MA). Rabbit polyclonal antibody against KRT14 (#PRB-155P) was purchased from Covance (Covance, Princeton, NJ). Rabbit polyclonal antibody against KRT4 (#HPA034881) was purchased from Sigma (Sigma-Aldrich Corp, St. Louis, MO). Rabbit monoclonal antibodies against E-cadherin (#3195), p75 (#8238), and phospho-histone H3 (#3377) were purchased from Cell Signaling (Cell Signaling Technology, MA). Mouse monoclonal antibody against p63 (#sc-8431) was purchased from Santa Cruz (Santa Cruz Biotechnology, TX). Mouse monoclonal antibody against PCNA (#MAB424) was purchased from Millipore (Billerica, MA). Goat polyclonal antibody against IL-33 (#AF3625) was purchased from R&D (R&D Systems, Minneapolis, MN). Donkey anti-goat Alexa Fluor 488 (A11055), anti-rabbit Alexa Fluor 568 (A10042), and anti-mouse Alexa Fluor 647 (A31571) secondary antibodies were purchased from Life Technologies (Carlsbad, CA). Mouse anti-HSP90 (TA500494) and mouse anti-GAPDH (TA310153) primary antibodies were purchased from Origene (Rockville, MD).

### Esophageal biopsy collection and processing

Esophageal biopsies were obtained and processed as previously described^43^. Briefly, this study was approved by the Institutional Review Board of Cincinnati Children’s Hospital Medical Center (CCHMC) before the start of the study. Active EoE was defined as having a physician-provided EoE diagnosis and ≥15 eosinophils per 400x high-power field in distal esophageal biopsies. Inactive EoE was defined as having a previous history of EoE but with 0 or 1 eosinophils per high-power field. Normal controls were defined as patients with any history of EoE nor any other eosinophilic gastrointestinal disorder (EGID) and 0 eosinophils per high-power field. After informed consent was received, distal esophageal biopsies were obtained and fixed with formalin and then embedded in paraffin (FFPE). All experimental methods utilizing processed esophageal biopsies were performed in accordance with all relevant guidelines and regulations.

### Immunohistochemistry and immunofluorescence of esophageal biopsies

Before immunohistochemistry or immunofluorescence was performed, hematoxylin and eosin (H&E) stainings of esophageal biopsies were examined to confirm proper orientation and inclusion of all layers of the epithelium. H&E stainings and immunohistochemistry of distal esophageal biopsies using mouse anti-IL-33 antibody (Nessy-1) were performed by the Pathology Research Core at CCHMC. Images were obtained using an Apotome widefield microscope (Zeiss, Thornwood, NY). For immunofluorescence studies, slides with 4-µm sections of FFPE esophageal biopsies underwent deparaffinization (serial incubations with xylene, 100% ethanol, 95% ethanol, 70% ethanol, 50% ethanol), antigen retrieval using sodium citrate buffer (10 mM sodium citrate, 0.05% Tween 20, pH 6.0), blocked with 10% donkey serum/phosphate-buffered saline (PBS), and then incubated with primary antibody diluted in 10% donkey serum/PBS overnight at 4 °C in a humidified chamber. The next day, slides were washed with PBS, incubated with secondary antibodies diluted in 10% donkey serum/PBS for 1 h at room temperature (RT) in a humidified chamber, and then washed in the presence of DAPI (0.5 µg/mL). Finally, a cover slip was added with ProLong Gold mounting reagent (Molecular Probes). The next day, slides were imaged using a Nikon A1R inverted confocal microscope. Analysis was performed with the Nikon Elements program.

### ***Ex vivo*** culture of primary esophageal epithelial cells

One human distal esophageal biopsy obtained during routine endoscopy was collected for research purposes in 1 mL keratinocyte serum-free media (KSFM) (Invitrogen) containing human epidermal growth factor (EGF) (1 ng/mL), bovine pituitary extract (50 μg/mL), and 1X penicillin/streptomycin (Invitrogen) and subsequently placed in a 60-mm dish in 3 mL of Leibovitz’s L-15 media (Invitrogen) containing 115 U/mL collagenase, 1.2 U/mL dispase, and 1.25 mg/mL bovine serum albumin that had been filter sterilized (0.2 μm). The biopsy was mechanically dispersed using scissors into pieces less than 1 mm in size and then incubated at 37 °C for 1 h. The digested biopsy was collected and washed twice with 5 mL KSFM containing the same supplements as described above. Cells were then incubated in 1 mL of 0.05% trypsin/ethylenediaminetetraacetic acid (EDTA) (Invitrogen) (10 min, 37 °C, with agitation every 2 min). Soybean trypsin inhibitor (250 mg/L in 1X Dulbecco’s phosphate-buffered saline) was added (5 mL). Cells were pelleted and then resuspended in 1 mL KSFM (containing the same supplements as described above) and transferred to a 35-mm dish. Irradiated NIH 3T3 J2 fibroblasts (162,500 cells) were added to the dish. Media were changed at day 5 and every other day thereafter using KSFM containing the same supplements as describe above. After epithelial cells became 60–70% confluent, they were dispersed from the plate using 0.05% trypsin/EDTA, which was inactivated by soybean trypsin inhibitor; cells were then cultured in KSFM containing the same supplements as described above. In indicated experiments, cultures were stimulated with recombinant human IL-13 or OSM (both from Peprotech).

### Immunofluorescence of primary esophageal epithelial cells

Primary esophageal epithelial cells were plated on Ibidi 8-well chambers (#80826). The next day, cells were fixed with 4% paraformaldehyde for 10 min and quenched with 50 mM ammonium chloride. Cells were blocked with 10% donkey serum/PBS for 30 min and incubated with primary antibody diluted in 10% donkey serum/PBS for 1 h at RT. Cells were washed with PBS, incubated with secondary antibodies diluted in 10% donkey serum/PBS for 1 h at RT, and washed in the presence of DAPI (0.5 µg/mL). Finally, cells were placed in fresh PBS and imaged using a Nikon A1R inverted confocal microscope. Analysis was performed with the Nikon Elements program.

### Western blot analysis

Cells were lysed in RIPA buffer (50 mM Tris-HCl pH 8; 150 mM NaCl, 1% Igepal, 0.5% sodium deoxycholate; 0.1% SDS, and 1 mM EGTA) supplemented with beta-mercaptoethanol and protease inhibitors (Roche), sonicated for three rounds of 10 seconds, boiled for 15 minutes, loaded onto a 4–12% SDS-PAGE gel (Invitrogen), and subjected to Western blot analysis. Membranes were probed with goat anti-IL-33, mouse anti-HSP90, or mouse anti-GAPDH antibodies. Secondary IRDye-conjugated antibodies were from LI-COR Biosciences (Lincoln, Nebraska). Quantification of signal was performed with Image Studio Lite software (http://www.licor.com/bio/products/software/image_studio_lite/).

### Quantitative real-time polymerase chain reaction (RT-PCR)

RT-PCR was performed as previously described^43^. Briefly, total RNA was isolated from cells using the RNeasy mini kit (Qiagen, Valencia, CA) according to the manufacturer’s protocol. RT-PCR was then performed using a 7900HT Fast Real-Time PCR system from Applied Biosystems (Life Technologies Grand Island, NY) with FastStart Universal SYBR Green Master mix (Roche Diagnostics Corporation Indianapolis, IN).

### Statistical Analysis

One-way ANOVA with Holm-Sidak correction for multiple testing was performed using GraphPad Prism 7.0 software.

## Electronic supplementary material

Supplementary Information
