# Supplementary material for: IL-33 is induced in undifferentiated, non-dividing esophageal epithelial cells in eosinophilic esophagitis
Source: Sci Rep. 2017 Dec 14;7:17563. doi: 10.1038/s41598-017-17541-5 (PMC5730585; doi:10.1038/s41598-017-17541-5)
Supplement: Supplementary file 1 — Supplementary Information [file 41598_2017_17541_MOESM1_ESM.pdf]

Supplementary Information

IL-33 is induced in undifferentiated, non-dividing esophageal epithelial cells in eosinophilic esophagitis

J. Travers, PhD[1#], M. Rochman, PhD[1#], J. M. Caldwell, PhD[1], J. A. Besse, BS[1], C.E. Miracle, BS[1], and M.E. Rothenberg, MD, PhD[1]*

[1]Division of Allergy and Immunology, Cincinnati Children's Hospital Medical Center, Cincinnati, Ohio 45229-3039, USA

# These authors contributed equally to this work

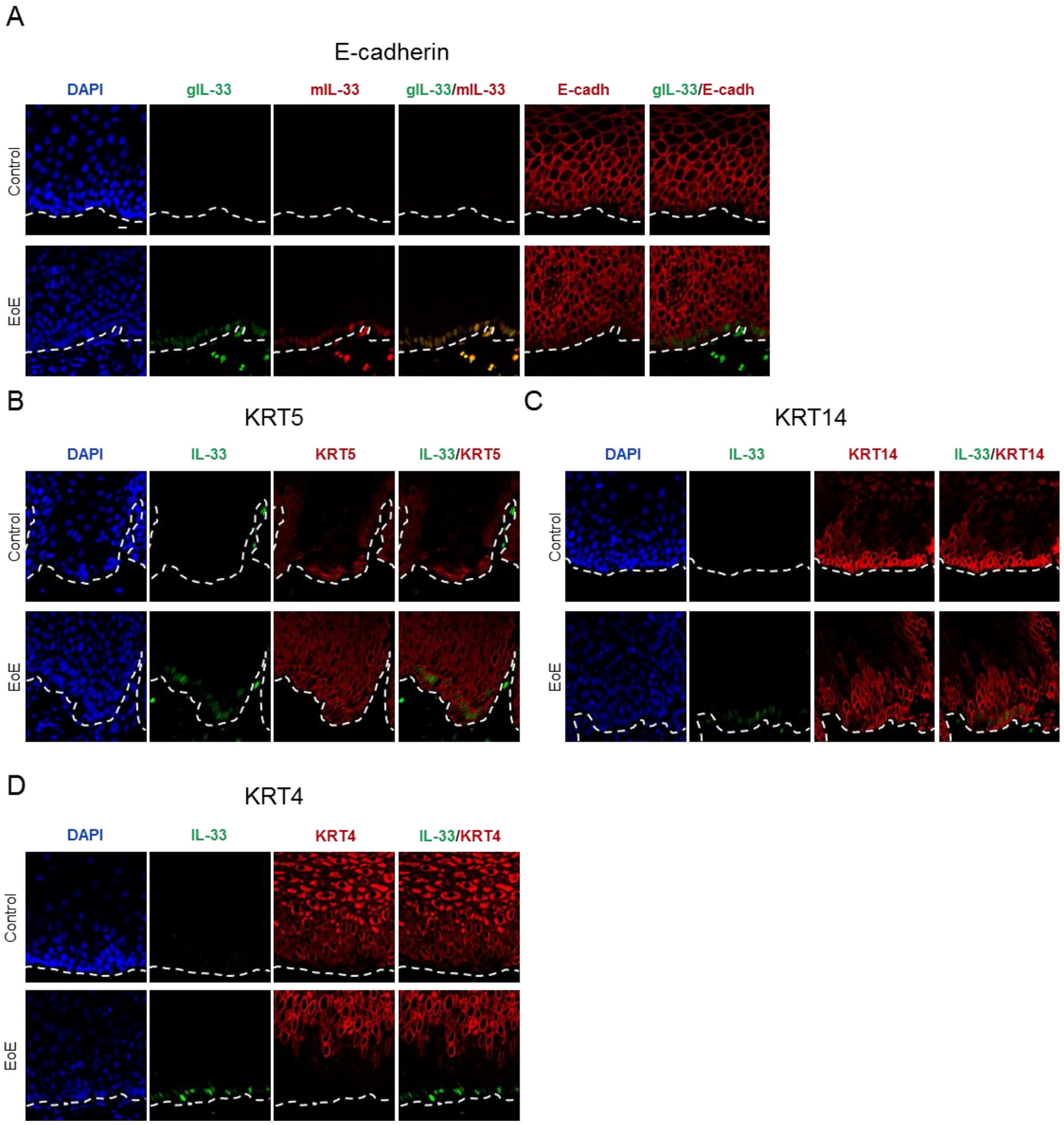

Supplementary Figure 1: Epithelium-specific protein expression in esophageal tissue. Low-power magnification images of the representative images from Figure 2 of immunofluorescence of esophageal biopsies from control individuals (top row) or patients with active EoE (bottom row). Nuclei are indicated by DAPI staining (blue). Green and red indicate staining with the

indicated antibodies. The white dashed lines indicate the basement membrane. Scale bar is 10 µm. Images are representative of biopsies from 4 or 5 patients with active EoE and 4 or 5 control individuals. DAPI, 4',6-diamidino-2-phenylindole; gIL-33, goat anti–IL-33 antibody; mIL-33, mouse anti–IL-33 antibody; KRT, keratin.

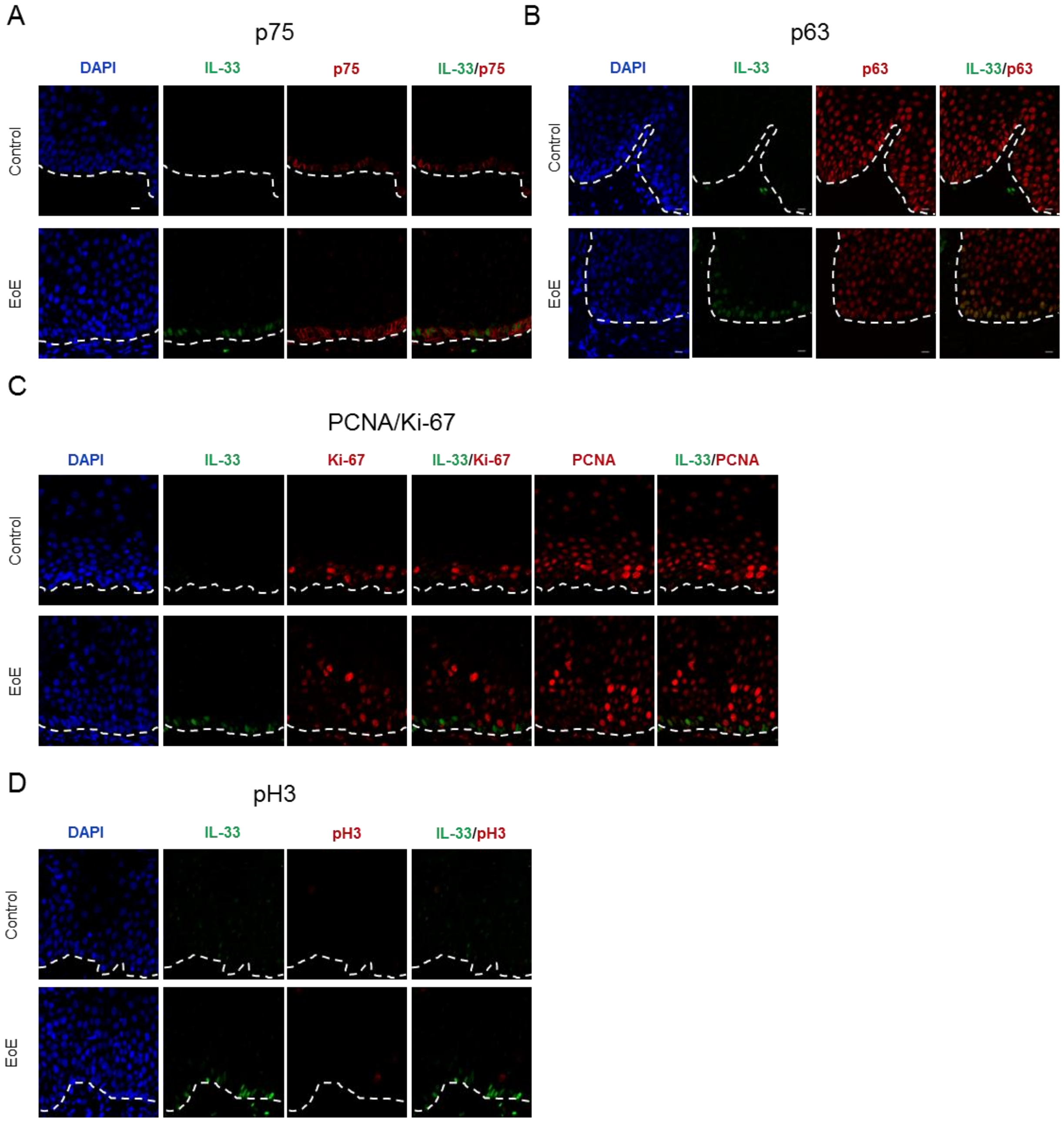

Supplementary Figure 2: Cell cycle and differentiation status in vivo in esophageal tissue. Low-power magnification images of the representative images from Figure 3 of immunofluorescence of esophageal biopsies from control individuals (top row) or patients with active EoE (bottom row). Nuclei are indicated by DAPI staining (blue). Green and red indicate staining with the indicated antibodies. The white dashed lines indicate the basement membrane.

Scale bar is 10 µm. Images are representative of biopsies from 3-6 patients with active EoE and 3-6 control individuals. DAPI, 4',6-diamidino-2-phenylindole; pH3, phospho-histone H3; PCNA, proliferating cell nuclear antigen.

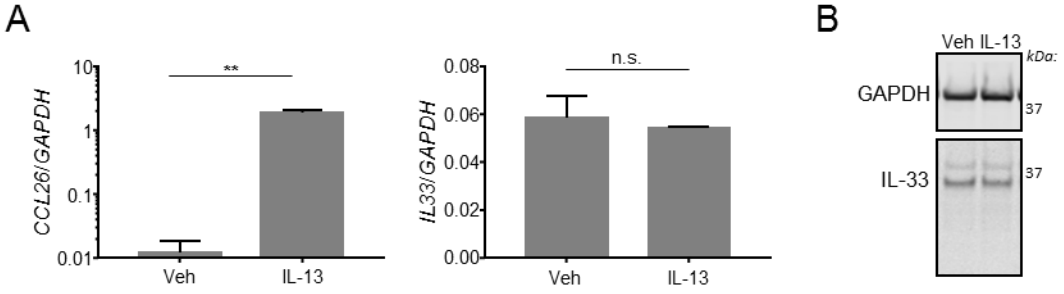

Supplementary Figure 3: Effect of IL-13 on IL-33 expression *ex vivo*. (A) Gene expression of *CCL26* (left) and *IL33* (right) in primary esophageal epithelial cells stimulated for 24 hours with 100 ng/mL of IL-13. Data is normalized to a housekeeping gene (*GAPDH*). Depicted are representative mean ± standard deviation of three independent experiments. (B) Western blot of analysis of IL-33 and GAPDH protein expression in primary esophageal epithelial cells stimulated with 100 ng/mL of IL-13 or vehicle for 24 hours. **, $p < 0.01$ and n.s., not significant using unpaired Student's t-test. CCL, chemokine (C-C) motif; GAPDH, glyceraldehyde 3-phosphate dehydrogenase; IL, interleukin; veh, vehicle.

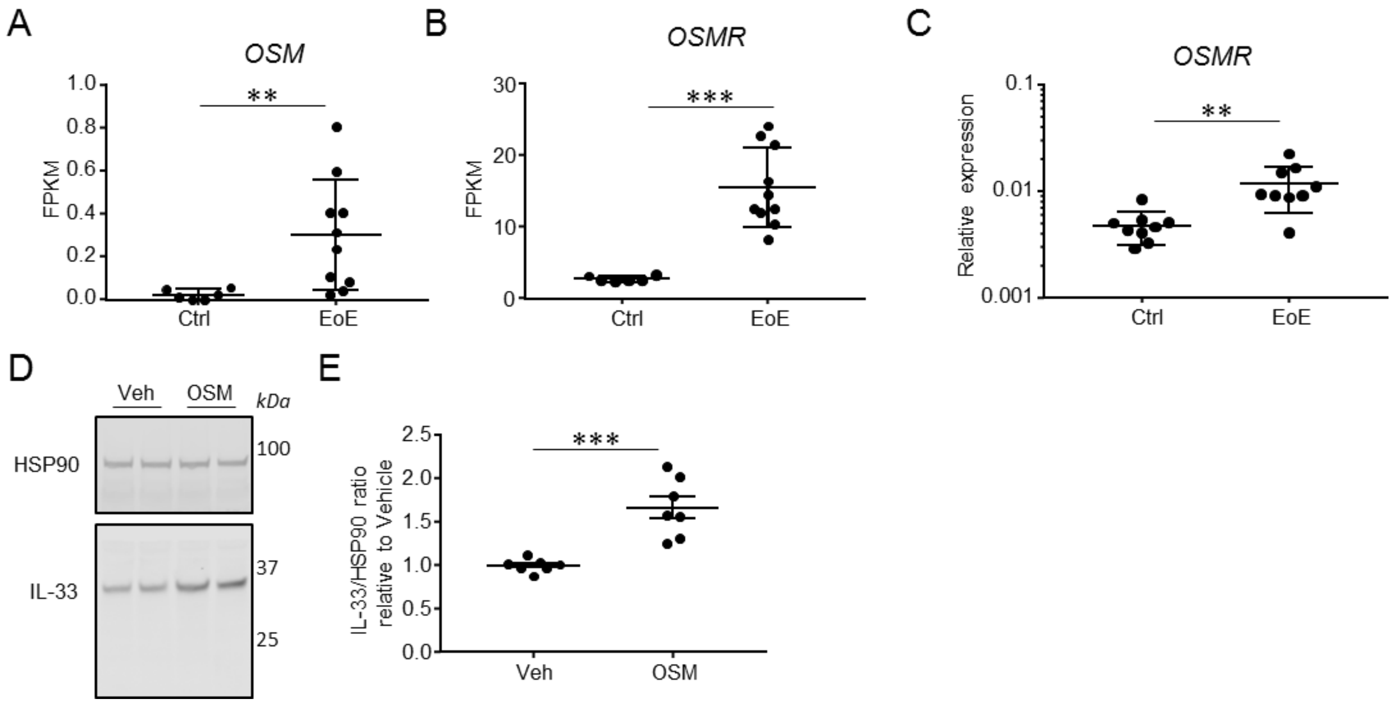

Supplementary Figure 4: Oncostatin M expression *in vivo* and effect on IL-33 expression *ex vivo*. (A-C) Gene expression of oncostatin M (*OSM*) or its receptor (*OSMR*) in esophageal biopsies derived from patients with eosinophilic esophagitis (EoE) or control individuals (Ctrl) as determined by RNA-sequencing[1] (A,B) or quantitative real-time polymerase chain reaction (C). Each dot represents an individual patient. Depicted are mean ± standard deviation. (D,E) Western blot of analysis of IL-33 and HSP90 protein expression in primary esophageal epithelial cells stimulated with 100 ng/mL of OSM or vehicle (Veh) for 24 hours with quantification in (D). Depicted are mean ± standard error of the mean of combined data from three independent experiments. **, $p < 0.01$ and ***, $p < 0.001$ using Mann-Whitney U-test. HSP, heat shock protein; IL, interleukin; kDa, kilodaltons.

REFERENCES

1 Sherrill, J. D. *et al.* Analysis and expansion of the eosinophilic esophagitis transcriptome by RNA sequencing. *Genes Immun* **15**, 361-369, doi:10.1038/gene.2014.27 (2014).
